# Maintaining a Sufficient Primary Care Workforce: A Problem We Should Not Have

**DOI:** 10.3389/fmed.2020.638894

**Published:** 2021-01-20

**Authors:** Arch G. Mainous

**Affiliations:** Departments of Health Services Research, Management & Policy and Community Health and Family Medicine, University of Florida, Gainesville, FL, United States

**Keywords:** primary care/general practice, setting of care, workforce (manpower), health policy, specialty choice, family medicine

Primary care has a positive impact on health equity, cost reduction, and improved quality of care (1–3). Primary care is characterized by the Cs of continuity, coordination of care, comprehensiveness and first contact care. Because of these characteristics, primary providers play a key role in the provision of care. For example, both patients and providers highly value continuity of care, a defining characteristic of primary care and a feature related to lower costs and better outcomes (2, 4–6). Consequently, strengthening primary care is a key policy strategy to improve the outcomes of health care delivery.

Unfortunately, there is a declining interest in primary care by US medical school graduates. Only 9% of US medical school graduates match in family medicine (7). The proportion of US graduates choosing family medicine has been low for many years, with family medicine residencies relying on international medical graduates. Further, complicating this is the fact that the US is that another source of primary care physicians that comes from post-graduate training is declining: general internal medicine practitioners, and general pediatricians (8, 9).

This issue of maintaining a sufficient primary care workforce isn't only a US problem. This is a worldwide problem. The proportion of general practitioners/family physicians is low or trending down in many countries ranging from Canada to Oman to France and is lower than that recommended by the World Health Organization (10–12). For example, in one study, only 20% of graduating students in France prefer general practice as their specialty choice (13).

## What are the Implications of an Insufficient Primary Care Workforce?

As was mentioned earlier, primary care has a positive impact on improved quality of care and cost reduction. An additional benefit that has substantial implications is access to care. For many health policymakers, access to care is synonymous with health insurance. Studies in the US commonly operationalize health care access as having insurance. That is a very limited view that doesn't pay justice to the value of primary care. In a country with universal access to health care, a place where everyone has health insurance, access to care shouldn't be a problem. A series of studies has shown that in the United Kingdom, in areas with low numbers of general practitioners, even though everyone has health insurance, health outcomes are worse (14, 15). In a study of all general practices in England, one of the strongest predictors of patient mortality was the number of GPs per 1,000 patients (15). That finding supports the case that general practice in England lacks capacity to meet demand and as a consequence, the patients do worse. Similarly, in a study of preventive primary care for children and risk of unplanned and ambulatory care sensitive condition (ACSC) hospital admission, a high uptake of preventive primary care from birth is associated with fewer unplanned and ACSC hospital admissions in children (16). These findings show that primary care access and a sufficient workforce is a key to providing quality health care.

Another clinical issue affected by an insufficient primary care workforce is the detection and management of chronic disease. Primary care providers are the first line for detection of disease and play an equally important role in the long term management of chronic disease. If there aren't enough primary care physicians for the population then detection and management of a common problem like hypertension falls through the cracks (14). The downstream implications are increased strokes, heart attacks and other complications from poorly managed hypertension.

We have seen during the COVID-19 pandemic that ACSCs like hypertension and diabetes play a huge role in the severity of illness with COVID-19 (17). Primary care plays a key role in health promotion and disease prevention, particularly with ACSCs. ACSCs and their management are dependent on access to appropriate care, and in particular primary care. In low income countries where the healthcare system is already weak, COVID-19 places a heavy burden and fractures care for chronic disease. It has been argued that appropriate primary care seems to play a positive role in decreasing the impact of COVID-19 and attention to conditions other than just flu-like illness may pay positive dividends (18).

In addition to the provision of clinical care, an insufficient primary care workforce has implications for the scientific base for primary care practice. Discussions about the need for more and better primary care research to improve practice have taken place for years (19). If the primary care workforce declines too low, then it is likely that the academic workforce will also decline. A likely consequence is that primary care research declines and the evidence based for the specialty declines as well. This could have significant deleterious implications for innovation and evidence based care in primary care.

## What Needs to be Done?

Maintaining a sufficient primary care workforce has positive benefits. The number of primary providers is inadequate for population health and effective delivery within health systems. The evidence is there for both of these assertions. We all want high quality care for patients and the population so how do we solve this ongoing and potentially worsening problem? First, from a structural standpoint in health systems, primary care needs to be emphasized. This is a position that needs to be embraced from a private health system perspective as well as a national health system perspective in countries around the world. Primary care physicians tend to be the lowest paid among medical specialties so an adjustment in compensation may need to take place. Discussion of a change in compensation across specialties will produce more than just a spirited discussion. It is likely to be met with substantial opposition. However, in the best interest of the patients and the population this difficult discussion needs to be had.

Further, it is important to think of all problems and solutions having a bit of a local flavor. Different countries will have different health structures and reimbursement systems. That said, the problem of maintaining an appropriate primary care presence cuts across many countries. There will likely need to be local solutions to this overarching problem.

In sum, we need to think creatively and act aggressively to maintain a sufficient primary care workforce. The evidence is there that solving this problem will be in the best interest of patients and the population.

## Author Contributions

AM conceptualized and wrote this article.

## Conflict of Interest

The author declares that the research was conducted in the absence of any commercial or financial relationships that could be construed as a potential conflict of interest.

## References

[1] Starfield B Shi L Macinko J Contribution of primary care to health systems and health. Milbank Q. (2005) 83:457–502. 10.1111/j.1468-0009.2005.00409.x 16202000 PMC2690145

[2] Bazemore A Petterson S Peterson LE Bruno R Chung Y Phillips RL Jr Higher primary care physician continuity is associated with lower costs and hospitalizations. Ann Fam Med. (2018) 16:492–7. 10.1370/afm.2308 30420363 PMC6231930

[3] Chang CH Stukel TA Flood AB Goodman DC Primary care physician workforce and Medicare beneficiaries' health outcomes. JAMA. (2011) 305:2096–104. 10.1001/jama.2011.665 21610242 PMC3108147

[4] Mainous AG 3rd Gill JM. The importance of continuity of care in the likelihood of future hospitalization: is site of care equivalent to a primary clinician? Am J Public Health. (1998) 88:1539–41. 10.2105/AJPH.88.10.1539 9772859 PMC1508474

[5] Oddone EZ Boulware LE. Primary care: medicine's gordian knot. Am J Med Sci. (2016) 351:20–5. 10.1016/j.amjms.2015.10.010 26802754

[6] Stokes T Tarrant C Mainous AG 3rd Schers H Freeman G Baker R. Continuity of care: is the personal doctor still important? A survey of general practitioners and family physicians in England and Wales, the United States, and The Netherlands. Ann Fam Med. (2005) 3:353–9. 10.1370/afm.351 16046569 PMC1466891

[7] American Academy of Family Physicians 2020 match results for family medicine. (2020). Available online at: https://www.aafp.org/medical-school-residency/program-directors/nrmp.html (accessed December 4, 2020).

[8] Dalen JE Ryan KJ Alpert JS. Where have the generalists gone? They became specialists, then subspecialists. Am J Med. (2017) 130:766–8. 10.1016/j.amjmed.2017.01.026 28216448

[9] Dall T Reynolds R Chakrabarti R Jones K Iacobucci W The complexities of physician supply and demand: projections from 2018 to 2033. Washington, DC: Association of American Medical Colleges 2020 The Complexities of Physician Supply and Demand: Projections From 2018 to 2033. Available online at: aamc.org (accessed December 4, 2020).

[10] Al-Salmani AA Al-Shidhani A Jaafar N Al-Mahrezi A. Factors associated with choice of career in family medicine among junior doctors in Oman. Sultan Qaboos Univ Med J. (2020) 20:e337–43. 10.18295/squmj.2020.20.03.014 33110650 PMC7574800

[11] Puertas EB Arósquipa C Gutiérrez D. Factors that influence a career choice in primary care among medical students from high-, middle-, and low-income countries: a systematic review. Rev Panam Salud Publica. (2013) 34:351–8. 24553763

[12] Scott I Gowans M Wright B Brenneis F Banner S Boone J. Determinants of choosing a career in family medicine. CMAJ. (2011) 183:E1–8. 10.1503/cmaj.091805 20974721 PMC3017271

[13] Lefevre JH Roupret M Kerneis S Karila L. Career choices of medical students: a national survey of 1780 students. Med Educ. (2010) 44:603–12. 10.1111/j.1365-2923.2010.03707.x 20604857

[14] Bankart MJ Anwar MS Walker N Mainous AG 3rd Baker R. Are there enough GPs in England to detect hypertension and maintain access? A cross-sectional study. Br J Gen Pract. (2013) 63:e339–44. 10.3399/bjgp13X667204 23643232 PMC3635580

[15] Baker R Honeyford K Levene LS Mainous AG 3rd Jones DR Bankart MJ. Population characteristics, mechanisms of primary care and premature mortality in England: a cross-sectional study. BMJ Open. (2016) 6:e009981. 10.1136/bmjopen-2015-009981 26868945 PMC4762103

[16] Cecil E Bottle A Ma R Hargreaves DS Wolfe I Mainous AG 3rd. Impact of preventive primary care on children's unplanned hospital admissions: a population-based birth cohort study of UK children 2000-2013. BMC Med. (2018) 16:151. 10.1186/s12916-018-1142-3 30220255 PMC6139908

[17] Garg S Kim L Whitaker M O'Halloran A Cummings C Holstein R. Hospitalization rates and characteristics of patients hospitalized with laboratory-confirmed coronavirus disease 2019 — COVID-NET, 14 States, March 1–30, 2020. MMWR Morb Mortal Wkly Rep. (2020) 69:458–64. 10.15585/mmwr.mm6915e3 32298251 PMC7755063

[18] Mainous AG 3rd Saxena S de Rochars VMB Macceus D. COVID-19 highlights health promotion and chronic disease prevention amid health disparities. Br J Gen Pract. (2020) 70:372–3. 10.3399/bjgp20X711785 32690480 PMC7376756

[19] Mullen R Weidner A Liaw W Mainous AG Hester CM Goodyear-Smith F Family medicine research capacity in the USA. Fam Pract. (2020) 7:cmaa119 10.1093/fampra/cmaa119 33159206

